# ACE-DNV: Automatic classification of gaze events in dynamic natural viewing

**DOI:** 10.3758/s13428-024-02358-8

**Published:** 2024-03-06

**Authors:** Ashkan Nejad, Gera A. de Haan, Joost Heutink, Frans W. Cornelissen

**Affiliations:** 1 Department of Research and Improvement of Care, Royal Dutch Visio, Huizen, The Netherlands; 2 Department of Clinical and Developmental Neuropsychology, University of Groningen, Groningen, The Netherlands; 3 Laboratory for Experimental Ophthalmology, University Medical Center Groningen, University of Groningen, Groningen, The Netherlands

**Keywords:** Gaze event classification, Mobile eye tracker, Machine learning

## Abstract

Eye movements offer valuable insights for clinical interventions, diagnostics, and understanding visual perception. The process usually involves recording a participant’s eye movements and analyzing them in terms of various gaze events. Manual identification of these events is extremely time-consuming. Although the field has seen the development of automatic event detection and classification methods, these methods have primarily focused on distinguishing events when participants remain stationary. With increasing interest in studying gaze behavior in freely moving participants, such as during daily activities like walking, new methods are required to automatically classify events in data collected under unrestricted conditions. Existing methods often rely on additional information from depth cameras or inertial measurement units (IMUs), which are not typically integrated into mobile eye trackers. To address this challenge, we present a framework for classifying gaze events based solely on eye-movement signals and scene video footage. Our approach, the Automatic Classification of gaze Events in Dynamic and Natural Viewing (ACE-DNV), analyzes eye movements in terms of velocity and direction and leverages visual odometry to capture head and body motion. Additionally, ACE-DNV assesses changes in image content surrounding the point of gaze. We evaluate the performance of ACE-DNV using a publicly available dataset and showcased its ability to discriminate between gaze fixation, gaze pursuit, gaze following, and gaze shifting (saccade) events. ACE-DNV exhibited comparable performance to previous methods, while eliminating the necessity for additional devices such as IMUs and depth cameras. In summary, ACE-DNV simplifies the automatic classification of gaze events in natural and dynamic environments. The source code is accessible at https://github.com/arnejad/ACE-DNV.

## Introduction

Analysis of visual scanning behavior is an essential component of several fields of research and has applied and clinical implications. The gaze changes that make up the visual scanning behavior are the result of eye-movements in combination with head motion. Applications range from human–computer interaction (Ohno et al., 2002) to diagnosis (Cesari et al., 2022; Crawford et al., 2005; Sweeney et al., 1991; Flechtner et al., 1997), rehabilitation (Gestefeld et al., 2020), education (Fichtel et al., 2019), biometrics (Holland & Komogortsev, 2013), and understanding fundamental aspects of visual perception and attention (Authié et al., 2017). One specific clinical application of eye movement and gaze analysis is in the field of neurovisual rehabilitation where, based on information on viewing behavior, therapists can intervene to teach more effective scanning strategies to their patients (Bouwmeester et al., 2007).

**Fig. 1:**
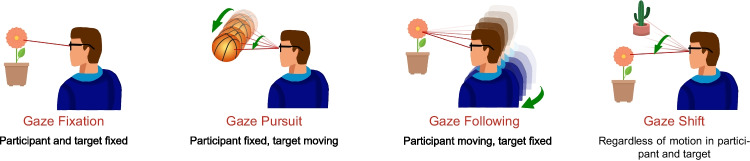
Illustration of the definition of gaze events in natural viewing proposed by Kothari et al. (2020)

Naturalistic viewing and scanning refers to the unconstrained and spontaneous visual exploration of the environment during everyday activities by means of dynamic and voluntary eye and head movements (Einhäuser et al., 2007). Understanding natural viewing is crucial for gaining insights into how humans interact with their surroundings, as it provides valuable information about the underlying mechanisms of visual perception and the integration of sensory information in real-world contexts.

Defining events in eye-movement behavior, such as fixations and saccades, is useful for distinguishing patterns in the behavior which may contribute towards understanding visual information processing and deployment of attention, amongst others. The analysis involves examining aspects such as the duration, distribution, and start and end points of specific events.

Mobile eye trackers are head-mounted, and while wearing them participants can perform daily living tasks that include body and head movements. Although stationary eye trackers can have higher sampling rates (Holmqvist et al., 2011), they cannot be used to record natural viewing, as these devices are generally limited to experiments with stimuli displayed or projected on screens. In recent studies of eye movements, mobile eye trackers have been used more and proved their performance (Hooge et al., 2022). In these natural viewing experiments, the gaze can be analyzed in a more realistic simulation of real-life tasks, providing information about scanning behavior in everyday life. However, allowing for head and body movements requires both adaptations to the definitions of the eye-movement events, as well as new methodologies to analyze the data.

### Eye-movement events

To quantify scanning behavior in stationary settings, researchers defined a set of events in the eye movements such as fixation, saccades, and smooth pursuits. According to a recent study by Hessels et al. (2018), researchers use various definitions for these events. Even though the choice of definition may depend on the specific application, Salvucci & Goldberg (2000) provided one of the most general ones: “fixations are pauses over informative regions of interest and saccades are rapid movements between fixations”. Smooth pursuit is defined as following a moving object with the eyes. These definitions of events only include eye-movements, while assuming a fixed position of the head and body and are used for head-centered or screen-centered reference frames.

By introducing head and body movement, it is essential to adapt the definitions. To ensure clarity in our use of specific terminology, we use the term gaze change for an eye movement coordinated with a possible change in head orientation (in a world-centered reference frame; Zangemeister & Stark (1982)). We employ the term eye movement when referring to the orientation and rotation of the eyes relative to the head (head-centered reference frame). Kothari et al. (2020) adapted the definitions of events by suggesting a set of events including gaze fixation, gaze pursuit, gaze following, and gaze shift, as illustrated in Fig. 1.

### Automated event detection/classification methods

Manually determining the eye-movement events in a recording is very time-consuming. For this reason, in the past decade, many event detectors and classifiers have been introduced for stationary settings. Salvucci & Goldberg (2000) used spatial and temporal information such as velocity, dispersion, and duration by setting static thresholds to identify fixations. Their findings were followed up by algorithms such as Identification of Velocity Movement Patterns (I-VMP) (Lopez, 2009; Larsson, 2016), and Identification of Velocity Dispersion-Threshold (I-VDT) (Komogortsev & Karpov, 2013) that could also classify smooth pursuit leading to ternary event classification. One of the disadvantages of these algorithms is that they strongly rely on the user’s choice of the static threshold (Komogortsev & Karpov, 2013).

Choosing this threshold depends on the eye tracker, task, and participant, which makes it hard to find a single threshold that can be used under all circumstances. Due to this limitation of static thresholding algorithms, researchers focused on designing methods that can dynamically adapt to different recording scenarios. For instance, Nystrom et al. introduced a settings-free method using an adaptive velocity threshold that outperformed the most commonly used algorithms at that time (Nyström & Holmqvist, 2010). Schweitzer & Rolfs (2020) proposed an improved adaptive two-dimensional velocity-based threshold for online saccade detection.

Further developments deployed machine learning to tackle this manual thresholding issue. Zemblys et al. (2018) used random forest algorithms for the detection of fixation, saccade, and post-saccadic oscillations. They evaluated their method on biometric data and showed that it outperformed previous methods.

In recent studies, researchers have turned to deep learning to improve classification accuracy. For example, U’n’Eye (Bellet et al., 2019) uses a convolutional neural network trained on horizontal and vertical velocity features to detect saccades, and was shown to outperform previous saccade detectors. Startsev et al. (2019) introduced an architecture using a 1D convolutional neural network followed by a bidirectional long-short-term network (1D-CNN-BLSTM) to enable a ternary classification of eye-movement events. Such a BLSTM network classifies events on the basis of time series rather than time points. As a result, its decisions are based on the pattern in the time series before and after the momentary time point being classified. Specifically, the method feeds the 1D CNN-BLSTM classifier a vector of features based on eye-movement velocity within multiple window sizes. The method’s results were shown to be more accurate and robust compared to the previous methods (Startsev et al., 2019).

**Fig. 2:**
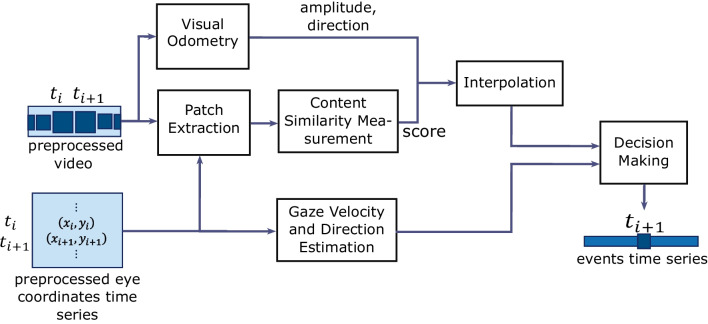
Illustration of the data pipeline of the ACE-DNV method on the cleaned data

Later, the method called Online Eye-Movement Classification (OEMC) used a temporal convolutional network (TCN) to further improve on 1D-CNN-BLSTM (Elmadjian et al., 2022). This method can be applied towards online event classification, as it relies only on previous time points to predict an event for the momentary time point. Whereas previous methods relied on preprocessed data, the extraction of predefined features or further post-processing steps, GazeNet (Zemblys et al., 2019) went one step further by classifying events from the raw eye-movement data. Nevertheless, its classification performance was at the level of expert human labelers.

Importantly, the GazeNet authors indicated that in natural viewing conditions, head movements would create a great challenge for event detection by both human labelers and hand-crafted algorithms (Zemblys et al., 2019). This issue presumably applies to all of the above-mentioned algorithms.

Thus, for detecting and classifying events in natural viewing conditions, a method should account for head movements. With mobile (head-mounted) eye trackers becoming ever more popular, this ability is becoming more essential. Thus far, only a few methods tackled gaze event detection in natural viewing conditions. Steil et al. (2018) took a video analysis approach, and extracted image information from small regions around the momentary gaze position (i.e., what falls on the central visual field). From this, their method computed similarity values based on which it could delineate the start and end of fixations. Since the method uses video information, which is commonly sampled at a relatively low rate (usually 30 Hz), this approach cannot detect rapid changes in events. Consequently, the approach of analyzing gaze region similarity, however valuable, is insufficient for ternary event classification.

In this study, we introduce a new method for the Automatic Classification of gaze Events in Natural Dynamic Viewing (ACE-DNV). It does so by using eye motion from the eye-movement signals and taking head and body motion into account while only relying on the scene camera video provided by modern mobile eye trackers. ACE-DNV computes the gaze and the camera motion and the similarity of the image content around the point-of-gaze. The extracted features are fed into a classification algorithm to determine gaze fixation, gaze following, gaze pursuit, and gaze shift. We show that our method is capable of learning and replicating manual labels. To the best of our knowledge, ACE-DNV is the first method able to classify these four natural viewing events by deriving head motion from scene video.

## Methods

In this section, we explain the building blocks of our method for event classification in natural and dynamic viewing and how these are connected as illustrated in Fig. 2. In addition, we discuss the used public dataset. Automatic Classification of Gaze Events in Natural Dynamic Viewing (ACE-DNV) uses recordings from mobile eye trackers and classifies each time point as being part of one of the four natural viewing events including gaze fixation, gaze pursuit, gaze following, and gaze shift.

### Gaze events in natural conditions

To define gaze events in real-life tasks and natural settings, we also need to take into account the movement in the head, body, and changes in the environment. In this paper, we use the definitions of events as used by Kothari et al. (2020) in the Gaze-in-Wild dataset:

1. During **Gaze fixation (GFi)**, the gaze of a stationary observer is stably aimed at a specific stationary object.
2. During **Gaze following (GFo)**, the observer keeps their gaze fixed on a stationary object while they themselves either rotate their head, or move their body from one location to the next.
3. During **Gaze pursuit (GP)**, a stationary observer is following a moving object using a combination of eye and head rotation. This is also referred to as smooth pursuit.
4. **Gaze shift (GS)** is a rapid gaze movement from one location to another.

In our study, we adopted the gaze event definitions as outlined in the paper by Kothari et al. (2020) that describes the Gaze-in-Wild dataset. This choice was primarily made to ensure consistency with this established dataset and comparability to this prior research. We selected four events that cover gaze behavior commonly observed in natural settings. We are aware that these four events will not capture every possible gaze interaction with static or moving objects and observers. The events are chosen as they help illustrate our framework’s capacity towards supporting the comprehension of gaze behavior in real-world contexts. As a consequence of this choice, our framework will only learn these types of events and not any undefined ones. It will thus not even consider the latter, and classify all data only in terms of the defined event categories. However, the flexibility of our framework allows other researchers to extend it by defining specific events tailored to their study objectives and to analyze more intricate scenarios.

By considering the given definitions, we postulate that the events can be categorized based on the changes in the eye-movement, head rotation, body translation, and image in the central visual field. An overview of the defined events is provided in Fig. 1.

Before starting the process, we remove the samples from the recordings associated with: (i) the eye tracker calibration, (ii) blinks, and (iii) any unlabeled sections of the recordings.

Video frames are passed into the DF-VO visual odometry method to extract the camera rotation and movement, reflecting head rotation and body translation. As a result, it produces a rotation vector in the form of quaternions and a translation vector, which are used to estimate the velocity of the translation in conjunction with the velocity and direction of rotation. In addition, a patch is extracted around each gaze location and fed into a content similarity measurement unit. The result of this process is a similarity score assigned to each of the two consecutive patches.

The velocity and direction of eye movement with a head-centered reference frame are concatenated with information from camera motion and gaze patch similarity as a feature vector and fed into the decision-making unit for training and testing purposes.

In the next subsections, we explain each unit in the pipeline in detail. The Feature extraction section discusses the feature extraction process including visual odometry, gaze motion, and gaze patch content similarity. The interpolation on the extracted features is explained in the Interpolation section. In the Within-event random sample extractor section, the within-event random sample extraction procedure is presented, which is used for data splitting and data balancing. The Decision-making unit section provides the specification of the used classification algorithm. We explain the training and testing procedure and our evaluation criteria in the Evaluation section.

### Feature extraction

As the first step, ACE-DNV extracts features from the eye-movement signal and the video of the scene camera. We show that classification of natural gaze events relies on quantifying the changes in eyes, head orientation, body location and the image content in the central visual field. We aim to create descriptive features based on the mentioned information to increase the discrimination capability.

**Fig. 3:**
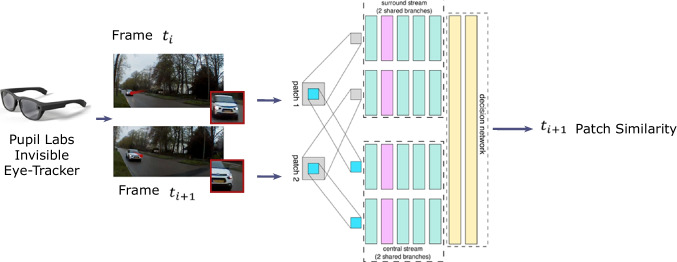
Illustration of the gaze patch similarity measurement using 2ch2stream network

#### Eye-movement features

The most popular feature in eye-tracking signal analysis is eye-movement velocity (Komogortsev & Karpov, 2013). It has been shown that the rotation of the eye can also provide information to detect smooth pursuit events (Authié et al., 2017) and gaze following events. In this paper, we make use of such information from the head-centered eye-movement signals collected by the mobile eye-trackers. In recent methods, the eye-movement velocity and direction have been computed with multiple sliding window sizes (Elmadjian et al., 2022; Startsev et al., 2019). In contrast, based on two consecutive samples (a window size of two) ACE-DNV calculates the eye-movement properties in terms of their velocity and direction. This helps in maintaining a lower number of features for faster classifications.

#### Scene gaze patch similarity

To eliminate the need for extra devices for the mobile eye-tracker, we extract the remaining features from the video of the scene camera. Our method projects the gaze locations on their corresponding video frames and extracts a surrounding patch of size 64-by-64 pixels. The gaze patches are fed into an already-trained deep neural network, named 2ch2stream (Zagoruyko & Komodakis, 2015), for similarity comparison of gaze area in video frames as suggested by Steil et al. (2018). 2ch2stream produces a score for the similarity of the consecutive patches. This provides us with information about the object being looked at by the participant. If the gaze moves from one object to another, the score similarity drops meaning that the image in the central visual field has changed. Figure 3 illustrates the extraction of gaze patches and how they are fed into the 2ch2stream neural network to obtain a similarity score.

**Fig. 4:**
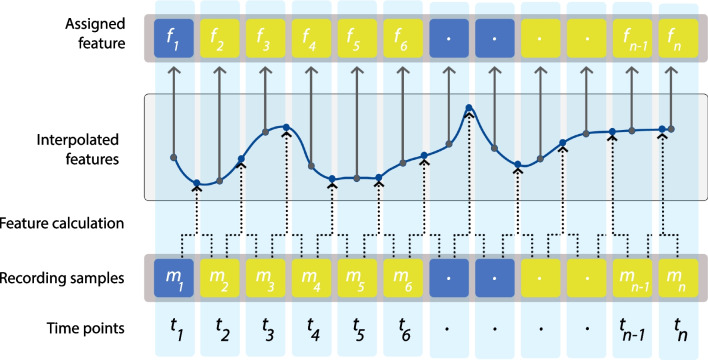
Assignment of features that are obtained from the differences of consecutive samples

Steil et al. (2018) evaluated the performance of such a similarity measurement for the purpose of fixation detection. They showed that performance based on the output of 2ch2stream is robust to the noise caused by head motion. Moreover, 2ch2stream allows detecting fixations in scenarios in which conventional methods fail.

#### Camera motion

Another feature extracted from the scene video is the camera motion. Kumar et al. (2022) showed that a visual simultaneous localization and mapping (VSLAM) method can better quantify the head rotation than an inertial measurement unit (IMU) for some natural viewing experiments. The core of a VSLAM method is a visual odometry (VO) unit which estimates the motion of the camera by analyzing the changes in the key points and the estimated depth in the videos. For a monocular method, the set of frames in a video recording is defined as $I=\{I_1, I_2, ..., I_n\}$. The objective of the VO method is to compute the relative transformation $T_k$ between video frames $I_k$ and $I_{k-1}$ (Scaramuzza & Fraundorfer, 2011). $T_k$ is formulated as follows:

$$\begin{aligned} T_k = \left[ \begin{array}{cc} R_k &{} t_k\\ 0 &{} 1 \end{array}\right] , \end{aligned}$$

where $R_k$ is the rotation matrix and $t_k$ is the translation vector.

ACE-DNV extracts the camera motion information by feeding the stream of frames into a monocular visual odometry method called DF-VO (Zhan et al., 2020). DF-VO produces a transformation information including a translation matrix and a rotation vector. The implementation code of this method is publicly available^1^. We define head rotation as the horizontal rotation of the camera, indicating changes in the observer’s viewpoint, while we define body translation as the camera’s movement within the environment, reflecting changes in the participant’s position, e.g. as occurring during walking. We compute the velocity and the direction of head rotation, and the velocity of body translation based on the output of DF-VO and include them in the feature vector.

### Interpolation

Since the sampling rate of the scene camera video is lower than that of the eye-movement signal, we perform interpolation to upsample the features extracted from the video. Hence, a function using cubic interpolation is fit separately to gaze patch similarity and camera rotation velocity, rotation direction, and translation velocity. At this stage, the signals scene camera and eye tracker are synchronized and cleaned for training and testing the decision-making unit.

Moreover, we utilize interpolation for features derived from the difference of two consecutive samples to estimate values for their specific time points. When dealing with two consecutive samples captured at time $t_i$ and $t_{i+1}$ with corresponding measurements $m_i$ and $m_{i+1}$, we extract a preliminary feature, assuming it corresponds to a time between $t_i$ and $t_{i+1}$. Each feature calculator receives the necessary measurements, including eye tracker signals and/or video frames.

Subsequently, through the interpolation of all the extracted features, we associate the exact time points with the function and derive approximate features $f_i$ and $f_{i+1}$ for $t_i$ and $t_{i+1}$, respectively (Fig. 4).

### Within-event random sample extractor

**Fig. 5:**
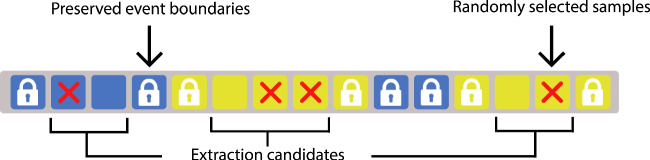
The within-event random sample extractor process used for balancing and data division purposes

In our approach, we used a within-event random sample extractor during some balancing and division steps. This function maintains the occurrence of events while splitting them into two subgroups of samples. We initiated the procedure by tallying the occurrence of each event across the target recordings distributed equally. Subsequently, we identified the number of occurring categories, and our selection process focused on extracting the prevalence of the other events to match the expected frequency. The process involved random selection of samples that were not located as event boundaries, minimizing potential impacts on future event-level scores. A specific extraction ratio was calculated for each category, and their samples were consistently extracted across all target recordings according to this ratio. The samples that were extracted can either be removed for balancing purposes or arranged into a new subset, preserving their frequency and order of occurrence in the original recordings while creating a set that can be used for testing. Figure 5 visualizes our within-event random sample extraction strategy.

### Decision-making unit

ACE-DNV utilizes a random forest (RF) (Breiman, 2001) classification algorithm because of its advantages for low-dimensional features and robustness to noise and overfitting. An RF ensembles multiple decision trees and trains each on a subset of samples. We used the random forest function provided by sklearn (Pedregosa et al., 2011) in Python. We executed this random forest function using its default values, as we found this resulted in the best scores.

### Evaluation

We assess and compare the performance of our method based on the F1 score at the event and sample level. An event-level evaluation consists of an event-matching strategy and a quality metric (Startsev & Zemblys, 2023). In this study, we employ the procedure proposed by Hoppe & Bulling (2016), which uses a majority voting event-matching strategy with F1-score quality metric. The F1-score is calculated based on achieved precision and recall. The mentioned terms are defined below:

$$\begin{aligned} Precision = \frac{TP}{TP + FP} \end{aligned}$$

$$\begin{aligned} Recall = \frac{TP}{TP + FN} \end{aligned}$$

$$\begin{aligned} F1= & {} \frac{(2 \times Precision \times Recall)}{(Precision + Recall)}\\= & {} \frac{TP}{TP + (FP+FN)/2} \end{aligned}$$

where TP, TN, FP, and FN are the number of true positives, true negatives, false positives, and false negatives, respectively. The F1-score reflects both correct and incorrect predictions providing a suitable measurement of the models. To determine the sample-level F1-score, we compare each individual sample predicted by the model on a continuous data stream against the actual data, while for event-level, the predicted event is chosen as the greatest frequency class among the predicted samples within a ground truth event.

As for the event-matching strategy, we treated consecutive labels as single events. A predicted event was determined as the class with the highest frequency within the ground truth span. This event-matching strategy is known as majority voting. This event-level scoring is generally more lenient compared to sample-level approach, as it does not heavily penalize failures in maintaining continuity for predicted events. Since this is a multiclass problem, we computed the true- and false-positive rates by means of one-vs.-all scoring and then obtained the macro-averaged results across all classes.

As the number of samples and types of events may vary in each test set, we calculate the weighted average of F1-scores. To do so, the relative frequency of occurrence of each event category in the test set was used as its weighting coefficients for the final average. Consequently, samples and events with a larger presence in the test set exert a greater influence on the overall average sample- and event-level F1-scores, respectively.

Our chosen event-level evaluation procedure as initially proposed by Hoppe & Bulling (2016) was recently used and implemented by Elmadjian et al. (2022). We used the evaluation function from their publicly available code^2^.

The distribution of the sample labels in an eye-movement recording may be highly imbalanced. This is due to the duration and occurrence of each type of event. For instance, saccades are of shorter duration compared to the other events. The event distribution also highly depends on the conducted task or activity by the participant during the recording.

To validate our method through training and testing on distinct subsets of the dataset, we employed two procedures: leave-one-out and train-test split. The subsequent subsections delve into the specifics of these procedures and outline their respective advantages.

#### Leave-one-out

The leave-one-out validation procedure serves to showcase the accuracy of the method on new recordings and in real-life applications. To achieve this, we isolate one recording within our target subset. The remaining recordings are balanced and input into the random forest for training. Subsequently, the isolated recording is utilized for testing. This process is iteratively performed, with each recording taking turns as the test set while the rest serve as the training set.

To balance the dataset, we employ an within-event random sample removal technique. Initially, we count the number of samples for each label across all recordings in the training set. To achieve balance, we decrease the number of samples in each category to match the category with the fewest samples, while maintaining the overall count of events. This is accomplished by utilizing our within-event sample extractor, as detailed in the Within-event random sample extractor section.

The drawback of leave-one-out lies in the variations between each iteration’s training sets. Due to differences between the training sets and the unbalanced test set in each iteration, explaining the underlying information learned by the random forest becomes challenging. This is mainly due to the differing numbers of samples and events in each iteration, making them non-comparable. To address this limitation, in addition to leave-one-out, we have also incorporated train-test split.

**Fig. 6:**
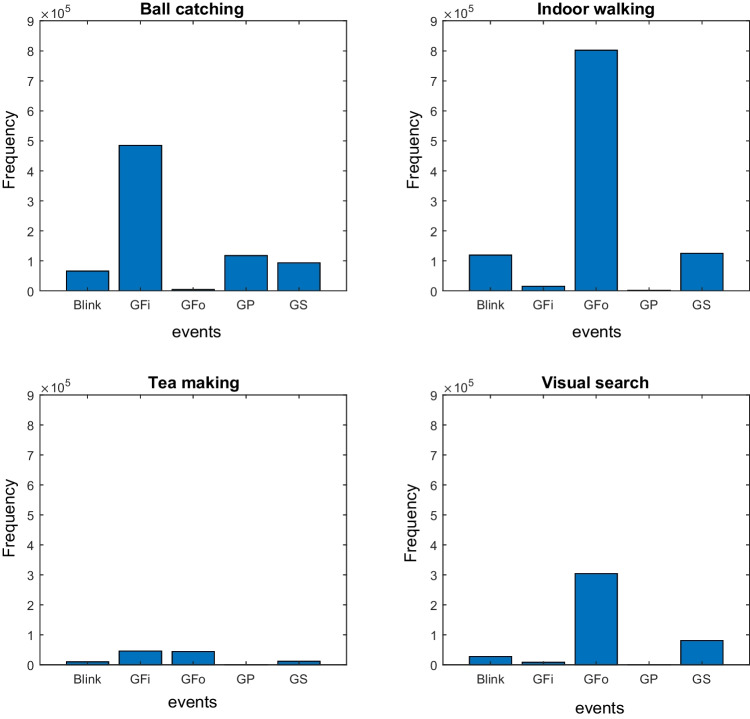
The distribution of the manually labeled events in four tasks of the Gaze-in-Wild dataset. There are substantial differences in the type and number of events made during the various tasks. Indoor walking, visual search, and tea-making include almost no gaze pursuit events since there are no moving objects in the environment. In contrast, ball catching includes gaze pursuit, but hardly any gaze following events since the participant does not move during the experiment. The difference in the total number of events is because of the difference in the number and length of recordings per activity

#### Train-test split

The train-test split, in addition to showcasing the classification performance, also offers explainability for the machine learning model. It provides insights into the underlying information in our data through features importance and the confusion among categories.

To split our time-series dataset into separate training and test sets while maintaining their temporal dynamics, we adopted the following approach: For the test set, we randomly extracted 20% non-overlapping samples within events and organized them in the same order of occurrence in the test set using our within-event random sample extractor explained in Within-event random sample extractor section. The remaining 80% of each recording was assigned to the training set. Figure 5 illustrates our event-preserving random sample extraction procedure.

Because the allocation of samples into the train and test sets is randomized, we observed potential inconsistency in case of random undersampling. To address this in the train-test split, we applied undersampling to all recordings by removing samples from the beginning of each recording, ensuring better consistency in the signals. To prevent events from the same category from attaching to each other and forming longer events, we marked the locations of removed events with a flag indicating a change of event.

It is essential to highlight that, for a comprehensive understanding of the machine learning method’s behavior, we aim for a normally distributed representation of categories in our samples. Hence, undersampling is carried out across all recordings, ensuring balance in train and test sets. This approach treats all events equally in terms of importance and persists same balance in all iterations of testing, resulting in consistent feature importance histograms and confusion matrices across iterations.

The RF feature importance histogram shows the achieved comparative importance of presented features to our RF model. This reports to what extent each feature plays a role in the classification process after being trained on the training process on the used subset of data.

The confusion matrix provides valuable information regarding the misclassifications. This helps in finding the events that are being mistaken by each other in the prediction process. The events in the eye-movement can be similar in nature resulting in misclassification among them. The confusion matrices help us determine the events that are being considered similar or the same by the model.

Our method captures the temporal dynamics of the samples during the feature extraction step which takes part before train/test division. We partition the dataset after feature calculation to prevent any disruption to the information found in consecutive samples. Since the classification does not work with temporal data and classifies at the sample-level, it does not result in data leakage between the train and test sets.

**Table 1:** The inter-labeler event-level F1 agreement in the Gaze-in-Wild dataset

Event	Agreement	
	Mean	Standard deviation
GFi	81	0.03
GFo	72	0.05
GP	71	0.05
GS	79	0.1
All	75	0.09

### Dataset

In this study, we used the Gaze-in-Wild dataset (Kothari et al., 2020) for training and testing our method. At the time of our study, this was the largest publicly available annotated natural viewing dataset. The dataset includes manually labeled recordings of participants conducting four different activities, which are indoor walking, throwing and catching a ball, visual search, and making tea. Multiple labelers manually annotated the data (Kothari et al., 2020). The dataset contains a total of 74 recordings, but not all were labeled. For the annotated recordings, the number of labelers varied from 1 to 4. Figure 6 shows the total number of labeled events for all labelers and all recordings in each task. The distribution of events in the recordings depends on the task.

As described in Kothari et al. (2020), the labelers were instructed to uniquely label each sample with one of six unique labels (zero to five), corresponding to unlabeled samples, fixations, gaze pursuits, gaze shifts, gaze followings, and blinks, respectively. Here, we limit our classification to the four labeled gaze events, by first discarding the samples labeled as blinks and unlabeled.

Table 1 reports the inter-labeler agreement in event-level F1-score by their mean and inter-subject standard deviation showing that the human labelers in this dataset have an average event-level F1 agreement of 75%. For this purpose, we executed the same procedure as conducted by Kothari et al. (2020), with considering gaze fixation and gaze following same events. However, we applied the event-matching strategy and quality metric proposed by Hoppe & Bulling (2016) for comparability.

## Experiments and results

In this section, we describe a series of experiments in which we assess the performance of ACE-DNV when it learned to predict the labels assigned by a single labeler to events in 1) a single task, 2) all tasks combined.

In our first experiment, ACE-DNV’s performance was assessed when trained and tested using the labels assigned by a single labeler in each specific task. For this, we selected the labeler with the most extensive labeling. In our second experiment, ACE-DNV was tested on all four tasks combined together, reflecting its generalizability to tasks encompassing diverse event categories. Additionally, in this experiment, we compared ACE-DNV’s performance against the Gaze-in-Wild classifiers. Additionally, we assessed feature importance using Random Forest histograms and calculated confusion matrices to gain insights into the crucial attributes influencing ACE-DNV’s predictions and scores.

We conducted our experiments using both leave-one-out and train-test split validation procedures. Leave-one-out offers insights into the method’s performance in real-life applications. However, due to variations in subsets of data in each iteration and the limitations of our dataset, the resulting feature histograms and confusion matrices differ in each iteration. In contrast, the train-test split, with its balanced and normally distributed subset of the data, allows us to report random feature histograms and confusion matrices, providing a clearer understanding of the learned information.

### Single labeler - single task

In our first experiment, we evaluated the performance of ACE-DNV in the case that it is trained and tested on the labels assigned by a single labeler to eye-movement events for a single task. Although the Gaze-in-Wild dataset (Kothari et al., 2020) contains four tasks, only three of these had sufficient recordings and labels to train and test our machine learning method. For each task, we chose the labeler that had assigned the largest number of labels. Labeler six did so for the ball catching (seven participants) and visual search tasks (three participants), while labeler five did so for the indoor walking task (eight participants).

Table 2 presents the obtained weighted average F1-scores for each class and the overall score using the leave-one-out validation procedure. Our findings indicate that ACE-DNV successfully predicted events such as indoor walking, ball catching, and visual search, achieving scores of approximately 74, 71, and 79%, respectively.

**Table 2:** Task-specific sample- and event-level classification performance (F1-scores) of ACE-DNV with leave-one-out procedure. For each activity, labels from the most contributing labeler are used

	Experiment 1						Experiment 2	
Task/Event	Indoor walk		Ball catch		Visual search		All tasks	
	Sample	Event	Sample	Event	Sample	Event	Sample	Event
GFi			69.6	70.8			58.7	49.5
GFo	81.9	70.7			85.3	80.3	60.6	67.3
GS	45.6	76.9	49.5	78.6	54.0	78.2	50.2	78.4
GP			25.7	23.4			20.6	17.1
Weighted Avg	76.3	73.8	61.4	71.0	78.8	79.2	56.8	67.8

**Table 3:** Task-specific sample- and event-level classification performance (F1-scores) of ACE-DNV with train-test split validation procedure. For each activity, labels from the most contributing labeler are used

	Experiment 1						Experiment 2	
Task/Event	Indoor walk		Ball catch		Visual search		All tasks	
	Sample	Event	Sample	Event	Sample	Event	Sample	Event
GFi			76.6	80.0			70.5	81.1
GFo	82.7	82.5			83.2	88.1	75.1	86.8
GS	82.1	83.0	77.6	84.5	82.4	88.0	72.8	86.7
GP			79.7	83.8			78.1	80.7
Weighted Avg	82.4	82.8	77.9	83.1	82.8	88.1	74.1	84.9

**Fig. 7:**
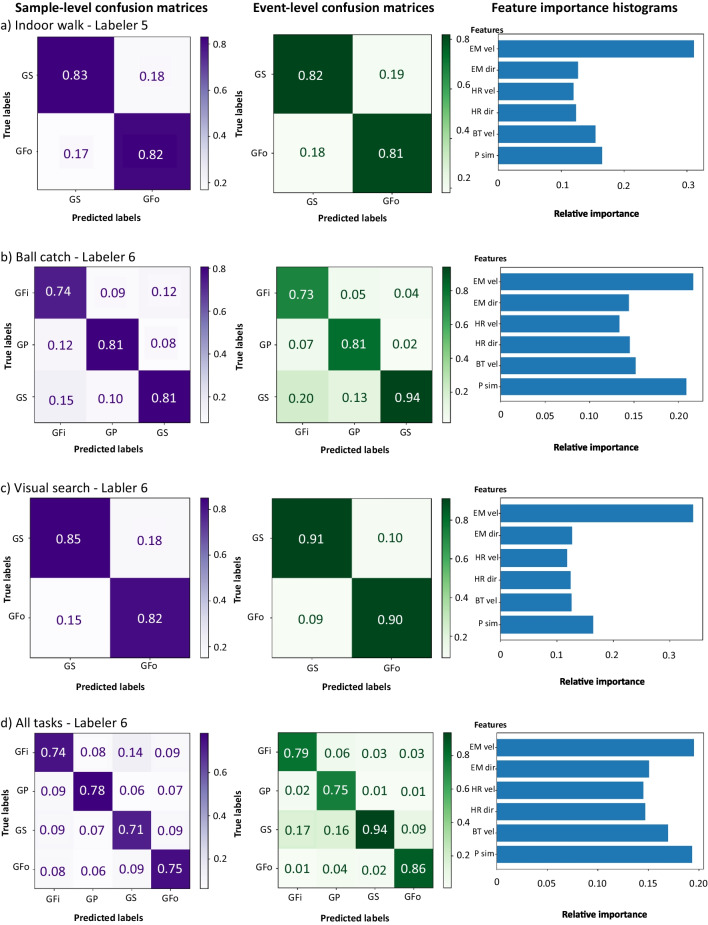
Confusion matrices for the event-level (*left panel*) and event-level (*middle panel*) prediction of gaze events. The *right panel* shows the feature importance histogram produced by the random forest model. On the right panel, the feature names from top to bottom represent, eye-movement velocity, eye-movement direction, head rotation velocity, head rotation direction, body translation velocity, and gaze patch similarity, respectively. Panels **a**, **b**, and **c** report our findings for training and testing our models on a single task using labels of the most contributing labeler for the tasks indoor walking, ball catching, and visual search, respectively. Panel **d** illustrates the performance of our model when it has been trained and tested on all the tasks combined

Table 3 shows the F1-scores for predicting the labels in the test sets. For indoor walking, ball catching, and visual search, ACE-DNV predicted events with scores of about 83, 83, and 88%, respectively. Its performance for different tasks was thus approximately similar. The confusion matrices shown in Fig. 7a–c indicate how often specific events were misclassified for each task. When predicting events in the ball catch task, in which three types of events occurred, ACE-DNV most often misclassified gaze shifts as gaze fixations and gaze pursuits.

In Fig. 7, the histograms (rightmost panels) show the feature importance for the random forest model in that experiment after training. It can be seen that eye-movement velocity was the most important feature for the indoor walk and visual search tasks. Note that in those tasks, only two types of events (gaze following and gaze shifting) occurred. In the ball catch task, three types of events (fixation, gaze pursuit, and gaze shift) had been observed. In this case, gaze patch similarity is almost as important as the eye-movement velocity for the random forest model.

### Single labeler - all tasks combined

In our second experiment, we tested the performance of our method in presence of all four events. This shows the generalizability of our method in case that it is executed on a new task in which all the four categories are present. For this purpose, the recordings from all four tasks labeled by labeler six were fed to our ACE-DNV with the undersampling process using our leave-one-out and train-test split validation procedures.

With leave-one-out validation procedure, in Table 2, we obtained total weighted average sample-level and event-level F1-scores of 57 and 67%, respectively. By comparing this experiment to the first experiment, gaze pursuit remains the most difficult category to predict in leave-one-out procedure.

As part of this experiment, we also compared the performance of ACE-DNV to that of the Gaze-in-Wild bidirectional recurrent neural network (BiRNN) classifiers shown in Table 4. For this comparison, it is important to note the difference between the Gaze-in-Wild methods and ACE-DNV. The Gaze-in-Wild method also uses signals derived from additional sensors and devices such as the IMUs and depth cameras. In contrast, ACE-DNV used only the scene video and eye-movement signals provided by current conventional mobile eye-trackers. Secondly, Gaze-in-Wild combined fixation and gaze following events into a single event type resulting in a three-way classification. For comparative analysis, we assessed our model by consolidating gaze following and gaze fixation into the same category during a leave-one-out validation procedure. For this experiment, we re-ran the Gaze-in-Wild BiRNN classifier using the metrics outlined in this manuscript.

Table 3 shows that ACE-DNV, in train-test split procedure, achieved sample-level and event-level F1-scores of about 74 and 84%, respectively. This indicates a slight decrease in its performance relative to Experiment 1, mostly likely due to the larger variation in activities and types of events. This may reduce the variation in this event resulting in a better classification. Figure 7d shows the associated RF feature importance histogram for this experiment. It resembles the one for the ball catch activity. This suggests that having pursuit events in the data reduces the singular importance of eye-movement velocity while the gaze patch similarity remains an important feature for the classification. The confusion matrices reveal that in this subset of data, gaze following was misclassified as gaze shift. However, through majority voting, the confusion was resolved in the event-level score but led to misclassification of gaze shift with the other events.

The BiRNN classifier of Gaze-in-Wild predicts with a weighted average of sample- and event-level F1-scores of 69 and 75%, respectively. The ACE-DNV shows performance scores of 64% for the sample-level and 73% for the event-level scores, respectively. This indicates that the performance of ACE-DNV was comparable to the Gaze-in-Wild classifier, but without requiring the signals from the IMUs and depth camera. The results show that gaze pursuit remains a challenging category for both methods.

**Table 4:** Event-level F1-scores for classification of ACE-DNV and the Gaze-in-wild classifiers with leave-one-out validation procedure

Event	Gaze-in-Wild - BiRNN		ACE-DNV	
	Sample	Event	Sample	Event
GFi+GFo	72.1	74.9	69.4	70.2
GS	75.1	80.1	71.5	79.4
GP	28.8	27.2	20.7	17.5
Weighted Avg	69.3	75.1	64.1	73.4

## Discussion

We have shown that it is feasible to automatically classify gaze events occurring during natural viewing tasks based only on the recordings and video provided by mobile eye trackers. As a major advantage over previous methods, our ACE-DNV framework only requires the eye-movement signals and scene camera video footage captured by current mobile eye trackers for automatically classifying four types of gaze events (fixation, gaze pursuit, gaze following, and gaze shifting).

Our choice and definition of events was based on those provided in the Gaze-in-Wild dataset (Kothari et al., 2020). The chosen events serve the primary purpose of showcasing the classification capabilities of our method, without an attempt at being exhaustive and all-encompassing. Researchers have the flexibility to tailor our framework to their needs; if they deem it necessary to add or replace event categories, this is possible, and the model can be retrained.

In our study, a challenge emerged from the integration of mobile eye-tracking technology and visual odometry due to their different reference frames. One the one hand, the eye-tracking data are head-centered, as it records eye movements relative to the observer’s head position and orientation. Also, the visual odometry method results are in a world-centered reference frame, in which motion and spatial coordinates are defined with respect to the external environment. This use of distinct reference frames requires careful consideration when interpreting and combining these data sources.

For classification of these gaze events, ACE-DNV uses a number of features including, eye-movement velocity and direction, head rotation velocity and direction, body translation velocity, and the similarity of the gaze patch content. ACE-DNV extracts these features from the eye-movement signals and scene recordings that current mobile eye trackers provide.

In a series of experiments, we examined ACE-DNV’s performance on a publicly available, labeled dataset. In the first experiment, we trained and tested our method using the labels of a single labeler. We showed that depending on the task during recording and the existing events, the event-level F1-score can range from about 74 to 79% in leave-one-out validation. The achieved scores in this experiment are higher than those in the second experiment. This indicates the expected performance when researchers would retrain and apply this method towards analyzing their own data and based on their own labeling of events. The resulting feature importance histograms also show that in the absence of gaze fixation and gaze pursuit, gaze velocity is more important. Nevertheless, while all four category of events exist, the similarity of content in the central part of the vision is as important as the eye-movement velocity.

While the data in the first experiment were limited to a single task, in the second experiment, we combined all tasks to investigate the performance of the method with more variation in the data. This showed the performance of the method in the case of learning labels from a single labeler on possibly more challenging tasks that a researcher would like to investigate. In the leave-one-out validation, ACE-DNV obtained an event-level F1-score of about 68%, with most misclassifications occurring for gaze fixations that were predicted to be gaze shifts. ACE-DNV’s performance is close to what can be considered maximally achievable with this particular dataset, given an total event-level inter-human-labeler agreement of 75%.

In the next step, we compared our method with Gaze-in-Wild’s BiRNN by testing both under identical conditions. Without making use of the signals from additional devices such as IMUs and depth cameras, our method performs at a similar level as the Gaze-in-Wild classifier. Moreover, ACE-DNV can predict gaze-fixation and gaze-following categories separately. For both classifiers, gaze pursuit was identified as the most challenging category.

The observation that gaze pursuit is a challenging category in the leave-one-out validation but not in the train-test split suggests that the model requires more exposure to this category during training. However, the presence of gaze pursuit in the train-test split in both experiments led to misclassification of gaze shift at the event level. Gaze pursuit events only occur in one of the conducted tasks (ball catch) in the dataset, with a low number of occurrences (as shown in Fig. 6). With a train-test split, the model could learn possible variations of gaze pursuit across all recordings. In contrast, isolating one recording in leave-one-out validation prevented the model from learning sufficiently from training participants to predict gaze pursuits for a new, unobserved participant.

Depending on the analyzed task conducted by the participants, the relevance of features tends to differ. For the indoor walk and visual search tasks, the participants were walking in the hallways of a building and looking at surrounding objects due to which only gaze following and gaze shift events occurred. In this case, eye-movement velocity is twice as important in the classification as the other features. However, for the ball catching task, which includes gaze pursuit, gaze fixation, and gaze shift events, this notable relevance of eye-movement velocity was reduced and gaze patch similarity became as important. When combining all activities and thus including different events, gaze patch similarity became the most important feature. This suggests the importance of content in the central part of the vision for classifying the gaze events.

## Limitations and future directions

At present, ACE-DNV does account for all possible changes in the environment that may affect performance score, such as changes in lighting or the presence of obstructions. Future work could consider these factors to improve ACE-DNV’s robustness. In the present study, we trained and tested ACE-DNV using the only available public dataset containing recordings obtained during natural viewing. Therefore, our current results are specific to this dataset. Future work should train and test ACE-DNV on additional datasets with more recordings. Also, the imbalance in the recordings and occurrence of events can cause a bias in the prediction models. Deployment of models more robust to such potential bias can improve the performance score without the need to discard or replicate the samples for balancing.

Moreover, in this project, for the train-test split, we balanced the entire recordings before dividing these into train and test sets to gain insight into the underlying information in the classifier. However, for the purpose of performance evaluation, a more accurate approach would be to balance only the training set. We used the present approach to avoid significant variations in training and testing iterations and to assign identical importance to all categories. However, future research could concentrate on evaluating ACE-DNV with more data to mitigate differences in training iterations.

For that balancing in the train-test split, we aimed to maintain data cohesion by removing samples from the beginning of the recordings, minimizing the impact on event-level measures. Nevertheless, it is crucial to consider event-level data balancing as a necessary future improvement for this type of work. However, in leave-one-out, the train sets were balanced using our within-event random sample extractor, while the test recordings in the leave-one-out procedure were not balanced. This approach was adopted to more reliably evaluate the performance of ACE-DNV.

Another aspect to consider is that ACE-DNV’s extracted motion and similarity features are relative estimates (relative to other video frames) and their magnitude cannot be directly translated to real-world quantities. This implies that if future studies would apply ACE-DNV to additional datasets, its model may need to be retrained. In particular, this would be necessary if the size or quality of video- or eye-tracking signals is different from what it has been trained on.

Even though the feature importance histograms show how informative each feature was for the classification, more detailed investigation will be necessary to gain a deeper understanding of this. For instance, for the gaze patch similarity, investigating the potential impact of noise in the videos could inform about the robustness of our method.

The method learns to reproduce an individual labeler’s perspective by incorporating their bias into its prediction. This may or may not be desirable. Training the method on consensus labels could remove such a personalized bias. Alternatively, future research may consider adding unsupervised classification of the events which may result in more rigid prediction of events.

We assessed the impact of subjective data labeling by executing our method on labels with consensus from two labelers. However, we observed inconsistencies between leave-one-out and train-test split results. Consequently, we presented the detailed experiment in the Appendix section.

## Conclusion

In conclusion, it is feasible to automatically classify gaze events made during natural viewing tasks based only on eye-movement data and video recorded by mobile eye trackers. Such a method can provide a potential solution for researchers and practitioners who are interested in analyzing scanning behavior in real-life tasks using gaze events. Our method is capable of classifying gaze events with a close to the inter-human labeler agreement scores, comparable with existing methods, and provides meaningful information on a given dataset. In addition, it is accessible to all users and can become a valuable tool for research and translational applications.

## Appendix A: Experiment 3: All tasks - agreement labels

**Table 5:** Sample and event-level F1-scores for testing on the agreement labels

Event	Train-test split		Leave-one-out	
	Sample	Event	Sample	Event
GFi	82.5	90.2	61.2	67.8
GFo	81.1	85.7	48.5	50.0
GS	86.7	93.0	51.8	88.5
GP	83.9	89.9	21.2	19.1
Weighted Avg	83.6	91.2	50.4	70.4

Manual labeling is a subjective task in which different biases and errors may affect the chosen labels (Hessels et al., 2018). Table 1 shows that this is also present in the Gaze-in-Wild dataset used in this paper. To somewhat overcome this issue, we extracted an agreement data subset for which at least two labelers agreed on the labels. This dataset allowed testing the ACE-DNV classifier on more consistently judged labels. In our third experiment, we evaluated our method on this subset of the dataset. This agreement data subset contained recordings from the indoor walking and ball-catching activities labeled by both labelers 5 and 6. In the entire dataset, labelers 5 and 6 labeled about 630,000 and 690,000 samples each, respectively, of which 235,000 samples were labeled by both of them. They agreed on the assigned event in about 170,000 of those samples.

Table 5 shows the event- and sample-level F1-scores on this agreement data subset in leave-one-out and train-test split validation procedures. The results indicate that classifying the subset with agreed-on labels appeared easier for ACE-DNV, showing a higher score in the train-test split scenario. The event-level score increased from about 85 to 91%, and the sample-level score rose from about 74 to about 83%. However, this observation does not necessarily hold in the leave-one-out validation procedure, where the scores are comparable to the second experiment.

It is crucial to note that the number of recordings is lower than in Experiment 2 (5 compared to 14), and, as mentioned, the number of samples and events is also lower, resulting in less variability. To achieve good scores in leave-one-out, the machine learning model needs to observe sufficient variability in the train set, enabling it to predict an unobserved entire recording. Hence, leave-one-out execution is more challenging in this experiment.

Our findings through the train-test split show that agreed-on labels simplify the task of classification, but leave-one-out does not demonstrate improvement. This experiment can benefit from further analysis on bigger datasets in the future.

## Footnotes

1 https://github.com/Huangying-Zhan/DF-VO

2 https://github.com/elmadjian/OEMC
